# Supplementary material for: Inflammatory Cytokines and ctDNA Are Biomarkers for Progression in Advanced-Stage Melanoma Patients Receiving Checkpoint Inhibitors
Source: Cancers (Basel). 2020 May 30;12(6):1414. doi: 10.3390/cancers12061414 (PMC7353055; doi:10.3390/cancers12061414)
Supplement: Supplementary file 1 [file cancers-12-01414-s001.pdf]

cancers

Supplementary Materials: Inflammatory Cytokines and ctDNA Are Biomarkers for Progression in Advanced-Stage Melanoma Patients Receiving Checkpoint Inhibitors

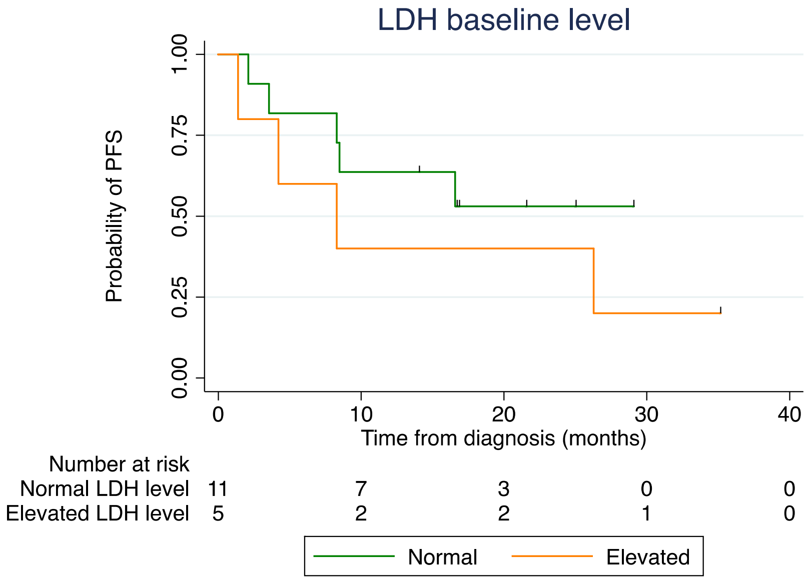

	LDH level
Log rank	
p-value	0.36
Cox regression	
HR (95% CI)	1.86 (0.48-7.27)
p-value	0.37

Figure S1. Survival analysis according to LDH level. Kaplan-Meier plot of progression-free survival (PFS) according to LDH level in the baseline blood sample (n = 16). Based on the LDH level, patients were divided into two groups; normal LDH level and elevated LDH level. HR; hazard ratio.

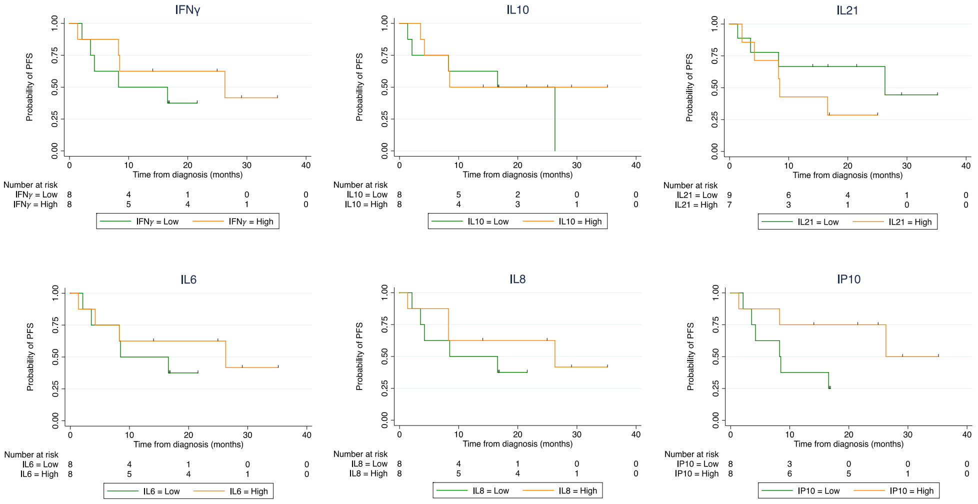

Figure S2. Survival analysis of baseline cytokine levels in patients treated with checkpoint inhibitors. Baseline plasma samples from 16 patients treated with checkpoint inhibitors were analysed for the concentration of interferon γ (IFNγ), Interleukin (IL) 10, IL21, IL6, IL8, and interferon γ-induced protein 10 (IP10). For each IFNγ, IL10, IL6, IL8, and IP10, the patients were dichotomized by the individual median cytokine concentration into a low and a high cytokine group. For IL21, patients were dichotomized according to undetectable (low) versus detectable (high) levels of IL21. Kaplan-Meier curves for progression-free survival (PFS) is shown for each of the six analysed cytokines. Tick marks denote censored patients.

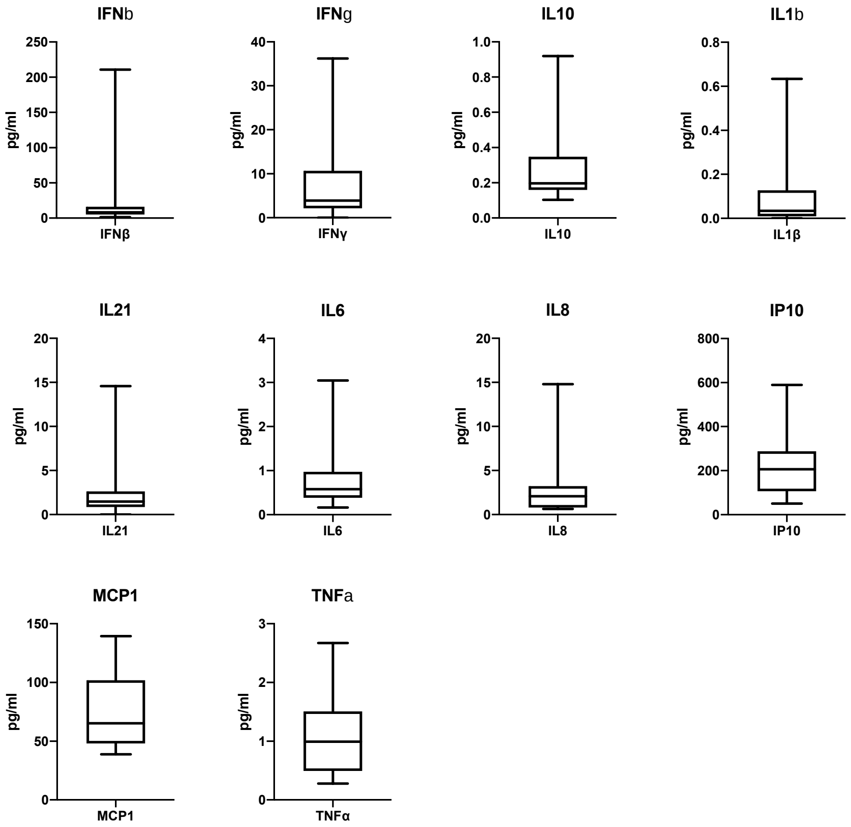

Figure S3. Interpatient cytokine variation. Box plots showing the concentration (pg/ml) of each cytokine. Each box represent the interquartile ranges, the central line indicates median, and the whiskers indicate the minimum and maximum values.

Table S1. ddPCR assay information.

Locus	Gene	Mutation		COSMIC ID	Bio-Rad assay ID	Amplicon size (bp)	Annealing temperature (°C)	Validated [1]	LoD (%)
		CDS	Amino acid						
Chr7:140453136	BRAF	c.1799T>A	p.V600E	COSM476	dHsaMDV2010027	91	55	Yes	0,1
Chr1:115256529	NRAS	c.182A>G	Q61R	COSM584	dHsaMDV2010071	65	55	Yes	0,2
Chr1:115256530	NRAS	c.181C>A	Q61K	COSM580	dHsaMDV2010067	65	55	Yes	0,2
Chr5:1295113	TERT	c.1-124C>T	N/A	COSM1716558	N/A[2]	116	62	N/A	0,3

Abbreviations: CDS, coding DNA sequence; del, deletion; *, translation termination; fs, frameshift; bp, base pairs; LoD, limit of detection; N/A, Not applicable; 1: Validated indicates if the assay has been wet-lab validated by Bio-Rad; 2: Designed and validated in-house; fwd primer: 5′-cct tca cct tcc agc tcc g-3′; Rev primer: 5′- gcc gcg gaa agg aag gg-3′; wild-type probe 5′-/HEX/cag ccc cct /ZEN/ccg ggc cct/IBFQ/-3′; Mutant probe: 5′-/FAM/cag ccc ctt/ZEN/ccg ggc cct/IBFQ/-3′; IBFQ: Iowa Black® FQ

Table S2. Lower limit of quantification for Meso Scale Discovery analysis.

Assay	Lower Limit of Quantification (pg/mL)		
	Plate 1	Plate 2	Plate 3
IFNβ	3,09	2,48	5,15
IFNγ	2,2	3,11	4,74
IL10	0,0658	0,0802	0,185
IL1β	0,0757	0,0767	0,227
IL21	3,92	0,914	3,06
IL6	0,209	0,115	0,272
IL8	0,089	0,095	0,378
IP10	0,43	0,702	1,03
MCP1	0,46	0,406	1,16
TNFα	0,496	0,462	0,829

Table S3. Contingency tables comparing ctDNA vs cytokine score, LDH vs ctDNA, and LDH vs cytokine score.

		Cytokine score	
		High	Low
ctDNA	Undetectable	5	3
	Detectable	0	4

Fishers exact test: $p = 0.0808$

		ctDNA	
		Undetectable	Detectable
LDH	Normal	6	2
	Elevated	2	2

Fishers exact test: $p = 0.55$

		Cytokine score	
		High	Low
LDH	Normal	3	5
	Elevated	2	2

Fishers exact test: $p = 0.9999$
